# Edição de Abril de 2022, vol. 118(42), págs. 797-857

**DOI:** 10.36660/abc.20220372

**Published:** 2022-06-06

Na “Diretriz Conjunta sobre Tromboembolismo Venoso – 2022”, com número de DOI: https://doi.org/10.36660/abc.20220213, publicado no periódico Arquivos Brasileiros de Cardiologia, 118(4): 797-857, foram realizadas as seguintes correções:

Incluída a instituição Hospital DF Star, Rede D’Or, Brasília, DF – Brasil, para a autora Simone Nascimento dos Santos.

Na instituição Angiolab Vitória, Laboratório Vascular, corrigida a localização “Rio de Janeiro, RJ” para “Vitória, ES”.

Na página 804, Quadro 2, foi inserida uma seta de “Positivo” para “USV”.

Somente na versão português, página 806, Quadro 5, coluna da direita, foi trocada a posição de “Positivo” e “Negativo”.

Somente na versão português, página 822, Quadro 9, linha 7, corrigida a grafia de “Compressibilidade”.

