# Supplementary material for: Edição de Abril de 2022, vol. 118(42), págs. 797-857
Source: Arq Bras Cardiol. 2022 Jun 6;118(6):1153. [Article in Portuguese] doi: 10.36660/abc.20220372 (PMC9345158; doi:10.36660/abc.20220372)
Supplement: Translation

# Translation: April 2022 Issue, vol. 118(42), pages 797-857

In the “Joint Guideline on Venous Thromboembolism - 2022”, with doi number: https://doi.org/10.36660/abc.20220213, published in the journal Arquivos Brasileiros de Cardiologia, 118(4): 797-857, the following corrections were made:

Included the institution Hospital DF Star, Rede D’Or, Brasília, DF – Brazil, for the author Simone Nascimento dos Santos.

At Angiolab Vitória, Laboratório Vascular, corrected the location “Rio de Janeiro, RJ” for “Vitória, ES”.

On page 804, Chart 2, an arrow from “Positive” to “VUS” was inserted.

Only in the Portuguese version, page 806, Chart 5, right column, the position of “Positivo” and “Negativo” was changed.

Only in the Portuguese version, page 822, Chart 9, line 7, corrected the spelling of “ Compressibilidade”.
